# The Tinnitus Handicap Inventory Total Score: What Really Counts? Experience on a Sample of 1156 Patients

**DOI:** 10.3390/audiolres15010004

**Published:** 2025-01-16

**Authors:** Roberto Teggi, Iacopo Cangiano, Marco Familiari, Vittorio Gioffrè, Alessandro Nobile, Omar Gatti

**Affiliations:** 1 ENT Division, IRCCS San Raffaele Scientific Institute, 20132 Milano, Italy; cangiano.iacopo@hsr.it (I.C.); nobile.alessandro@hsr.it (A.N.); gatti.omar@hsr.it (O.G.); 2 ASST Nord Milano, Sesto San Giovanni, 20099 Milano, Italy; familiarimarco@gmail.com

**Keywords:** tinnitus, THI, anxiety, migraine, hearing threshold

## Abstract

Background: Tinnitus is a frequent symptom, and is present in 10–15% of people who suffer from chronic tinnitus, defined as heard every day for at least 6 months. Among these, 1–2% develop a strong emotive reaction, anxiety, and depression, leading to poor quality of life. Objectives: to evaluate the comorbidities in tinnitus sufferers. Methods: In our retrospective study, we collected data on 1156 subjects with tinnitus present for at least 3 months, including age, audiometric exam, THI questionnaire, vascular disorders, fluctuations, causal factors, lifetime psychiatric disorders, and the presence of migraine. A linear regression model was used to assess the independent role of these variables on the THI total score representing tinnitus annoyance. A lifetime history of psychiatric disorders and migraine were predictive for the development of a disabling tinnitus. Results: Among comorbidities a history of previous psychiatric disorders was predictive for developing tinnitus. Moreover, no correlation has been found between hearing level and THI total score. Conclusions: Our data are not inconsistent with the hypothesis that psychological disorders and a particular personality trait may be the main causal factors for tinnitus annoyance.

## 1. Introduction

The term subjective tinnitus refers to a sound heard by a patient in the ear/s or head, but which does not exist in the outer world [1]. It is far from being rare and it has been estimated that 10–15% of people suffer from chronic tinnitus, defined as heard every day for at least 6 months [2]. Among these, 1–2% develop a strong emotive reaction, anxiety, and depression, leading to poor quality of life [3]. Psychiatric disorders in tinnitus sufferers are not rare, and according to some authors, a comorbidity is present in around 20% of subjects [4]. On the other hand, it is still under debate whether a peculiar personality trait, defined as a characteristic pattern of thinking, feeling, and behaving of an individual, may contribute to the development of severe tinnitus. Some authors have proposed that persons with a distressed personality trait prone to neuroticism develop tinnitus more easily, and, when present, it has a more severe impact on their quality of life [5,6,7,8].

It is commonly accepted that the generation of tinnitus in many individuals is associated with decreased input to the auditory system caused by hearing loss [9].

In several papers, Jastreboff proposed a neurophysiological model of tinnitus, which integrates neurophysiological and psychological features. It includes predisposing, precipitating, and maintaining factors; tinnitus may be generated as a consequence of hearing loss, possibly recovered, but its maintenance and distress are a consequence of circuit malfunction in a neural network, involving sensory, limbic, and autonomic components [10,11]. The model, separating the generation of tinnitus from maintenance and annoyance, can also explain the high proportion of tinnitus sufferers with a normal level of hearing [12]. It can also explain PET and EEG findings in these subjects, showing anomalies in central and limbic structures even when there is a normal level of hearing [13,14].

In the clinical assessment of tinnitus, questionnaires have a central role; among them, the Tinnitus Handicap Inventory (THI) is probably the most widely used. First proposed by Newman and Jacobson in 1996, the self administered survey consists of 25 items. Each item has three potential answers, with “yes” assigned 4 points, “sometimes” 2 points, and “no” 0 points. This leads to a total score ranging from 0, indicating no tinnitus handicap, and 100, indicating the worst patients’ annoyance. It is composed of three subscales: a functional (12 items) subscale, an emotional (8 items) subscale, and a catastrophic subscale (5 items) [15].

A grading system has also been proposed by the British Association of Otolaryngologists, Head and Neck Surgeons, based on five degrees of severity measured with the THI total score [16]:

- Very mild (score 0–16). Tinnitus is perceived only in silence and is easily masked. It does not interfere with sleep or with daily activities.
- Mild (score 18–36). Tinnitus is easily masked by environmental sounds and forgotten during daily activities. It can occasionally interfere with sleep, but not with daily activities.
- Moderate (score 38–56). Tinnitus is perceived even in the presence of environmental sounds; however, daily activities are not impaired. It is perceived less under concentration. Interference with sleep and relaxing activities is not infrequent.
- Severe (score 58–76). Tinnitus is continuously perceived and hardly masked by external noise. It alters the sleep cycle and can interfere with the subject’s daily activities. Relaxing activities are compromised. Subjects with this level of tinnitus often require medical consultations.
- Catastrophic (78–100). All side effects caused by tinnitus are present at a very severe level. The subject requires medical assistance very frequently, including neuropsychiatric help.

An Italian validated version of the THI questionnaire was published in 2008 [17].

## 2. Materials and Methods

In this retrospective study, we collected data on 1156 subjects with tinnitus present for at least 3 months; data were obtained from records of patients who visited our tinnitus outpatient clinic from 2007 to 2022. All patients underwent an audiometric exam and completed the THI questionnaire. All audiometric exams were performed by the same audiometrist in a soundproof chamber including frequencies between 125 and 8000 Hz.

According to the audiometric exam, they were grouped into four categories:

- Normal hearing level;
- Symmetric decrease in hearing level on acute tones;
- Decrease in hearing level on acute tones with a gap between the two threshold of at least 30 db on two contiguous frequencies;
- Monolateral hearing loss without fluctuation.

The following data were also collected:

- If tinnitus was provoked by any cause;
- Previous vascular disorders of the heart or central nervous system;
- Lifetime psychiatric disorders for which they were evaluated by a psychologist/psychiatrist;
- Presence of migraine according to International Headache Society (HIS) criteria [18];
- If tinnitus presented fluctuation on specific days;
- If tinnitus was present bilateral, unilateral, or in the head.

All patients presenting an asymmetric hearing loss underwent a central nervous system MRI to rule out acoustic schwannomas.

Exclusion criteria included the following:

- Previous vertigo episodes of any kind;
- Ongoing therapy for tinnitus, migrainous headache, or psychiatric disorders;
- Ongoing psychological therapies;
- Ongoing therapies for neoplastic lesions or central nervous system disorders other than vascular disorders;
- Lifetime history of vertigo.

### Statistical Analysis

Continuous variables are reported as mean and standard deviation. Categorical variables are presented as total number and percentage. A *p* value ≤ 0.05 was considered statistically significant. An ANOVA for repeated measures was performed to assess differences in continuous variables; a chi square test was used to assess differences in categorical variables. A linear regression model was calculated to assess the independent role of age, migraine, the clinical history of vascular disorders of CNS, hearing loss, and the fluctuation of the tinnitus on the THI total score. A Spearman test was performed to assess correlation between variables.

## 3. Results

The total sample was composed of 1156 subjects; 508 were females (43.9%).

The mean age at consultation was 54.1 ± 15.7. Sixty-three subjects (4.5%) reported that their tinnitus was present for less than 6 months, 378 (33%) from 6 to 12 months, and the remaining 715 (61.8%) reported it being present for more than 1 year. The mean value of the THI questionnaire was 43.1 ± 24.

No difference was detected between THI values in females (44.7 ± 22.1) and males (42 ± 22.8, *p* = 0.08). Similarly, no difference was detected in THI total score between subjects with the onset of tinnitus within 1 year (41.9 ± 27.5) and over 1 year (44.3 ± 24.2; *p* = 0.06).

When asked about the causal factor, 6 (0.5%) replied that it occurred after head trauma, 1 occurred after meningitis, in 155 (13.4%) subjects it occurred after a period of hearing loss for any reason, in 76 (6.6%) subjects the onset was during a stressful period, while 918 (79.5%) subjects were unable to recognize a causal factor.

In the cohort, 474 (41.1%) subjects reported bilateral tinnitus, 146 (12.6%) perceived it as being in the head, 338 (29.2%) reported it on the left, and 198 (17.1%) on the right ear.

Among comorbidities, 218 (18.8%) subjects reported a lifetime period of anxiety/depression for which they underwent psychiatric therapies, 118 (10.2%) were migraineurs, 135 (11.7%) were undergoing therapy for myocardial ischemia, and 85 were undergoing therapy for vascular disorders of the central nervous system.

The THI total score and total number and percentage of patients belonging to the different gradings of the tinnitus in the subgroups of subjects with the normal hearing level, symmetric hearing loss, asymmetric hearing loss, and monolateral hearing loss are summarized in Table 1. An ANOVA test detected no difference between groups; a chi square test showed no difference for the grading of the THI score between groups. A Spearman test detected a correlation between age and hearing loss (*p* = 0.03).

A linear regression model considering age, recognized causal factor, fluctuations in the tinnitus, migraine, a clinical history of vascular disorders, and hearing loss as independent variables, and THI total score as a dependent variable, has been performed. Results are shown in Table 2. A Spearman test demonstrated a correlation between psychiatric disorders and migraine (rs 0.18, *p* = 0.02).

Finally, we assessed the THI grading in the subgroups of subjects with and without previous psychiatric therapies; a chi-square test demonstrated a higher annoyance in the group with a history of diagnosed psychiatric disorders (chi-square statistic is 47.6, *p*-value is <0.00001). Results are summarized in Table 3.

## 4. Discussion

The neurophysiological model of tinnitus proposed by Jastreboff [10,11] suggests that tinnitus may result from the abnormal processing of a signal generated in the auditory system. Therefore, hearing loss provoking pathological changes in the cochlear neurotransmission and auditory pathways can in turn be a factor in the development of tinnitus; studies on animal models seem to confirm this theory, since exposure to noise or use of ototoxic drugs often results in the appearance of tinnitus [19]. On the other hand, abnormal processes of the signal generated within the auditory pathways through links with the limbic system may create a loop system, increasing the annoyance of the tinnitus, which in turn increases attention even when the hearing loss regresses. The relationship between tinnitus and the activity of the limbic system, which mediates emotions, has been widely studied. It can be summarized that researchers hypothesize that persisting tinnitus plays a “central” role of selective attention and dysfunctional cognitive processing [20,21]. This model may explain why some patients may be so distressed about the tinnitus and why in some cases they experience a drastic reduction in their quality of life [22,23]. It is still under debate as to whether a particular personality trait, named type D, in which individuals are more prone to neuroticism, may act as a predisposing factor for tinnitus [24].

In order to evaluate the annoyance of tinnitus, Newman and Jacobson proposed the THI questionnaire in 1996 [15]. The THI was shown to be a robust, psychometrically adequate measure of the impact of tinnitus on everyday life and annoyance [25,26]. Recently, an Italian version of the questionnaire has been validated [17].

Our sample was composed of patients with chronic tinnitus with an onset of at least 3 months; since the recruitment of patients was made in a second/third level outpatient clinic, our data may be representative mainly of a proportion of tinnitus sufferers with high annoyance and can present specific features.

Firstly, as in other reports, we were unable to detect a correlation between hearing level and THI total score [27,28]. Controversial results have been published on this topic [29]. The onset of tinnitus is strongly correlated with hearing loss, although it is unclear why some people with hearing impairment develop tinnitus and others do not. It must be noted that in our sample, only 31.4% had a normal hearing level, while annoyance did not differ between normoacousic subjects and those with hearing impairment. In particular, a severe tinnitus was equally represented in normoacousic subjects and patients with hearing impairment; these results are in line with those published by other authors, reporting a mild to no association between hearing loss and annoyance [30].

In contrast, we found a mild correlation between age and THI total score. Controversial results have been published on this aspect [31,32,33]. Curiously, it should be noted that in our sample, the elderly more often presented hearing impairment, but the latter variable did not correlate with THI score; among other possibilities, it should be considered that the elderly are more prone to anxiety and depression, which are probably major factors for tinnitus annoyance [34,35].

Finally, we found that the presence of previous psychiatric disorders was highly predictive in developing an elevated THI total score. These results are in line with many previous works. Tinnitus is often accompanied by anxiety and depression and previous studies on anxiety and depression found that 48% of sufferers presented various degrees of depression [36]. Another investigation studied the psychosocial stressors in tinnitus sufferers; they reported that the majority (77%) had drastic financial, marital, or sibling stressful life events [37].

Based on these data, some authors suggested that the psychological stressor greatly impacts physiological function with improper stimulation of the autonomic nervous system [38].

The findings support Jastreboff’s model, reporting that perceived tinnitus does not evoke annoyance and highlights the importance of emotional processing as a major factor in generating tinnitus distress [10].

It is under debate as to whether a specific personality trait is a predisposing factor to tinnitus complaints or mainly aggravates symptoms. Many studies have investigated this point. In particular, several studies have examined the correlation between tinnitus annoyance and neuroticism. Neuroticism is the trait disposition to experience negative affects, including anger, anxiety, self-consciousness, irritability, emotional instability, and depression; normally these subjects respond poorly to stressful situations and interpret ordinary situations as threatening [39]. Different authors proposed a correlation between this personality trait and tinnitus distress [40,41], with it being the first a predictor of the onset of tinnitus [42] and increased annoyance. Although we are unable to correlate the age of onset of tinnitus in our sample and the period of psychological distress, we believe that our data are not inconsistent with the previously reported hypothesis.

Other authors published data on an increased prevalence of migraineurs among tinnitus sufferers, and patients with comorbid headaches have higher scores on a tinnitus questionnaire, a lower quality of life, and more frequent comorbidities such as a painful sensation to loud sounds [43]. In our sample, we found a correlation between migraine and psychiatric disorders which can explain our data. The association between migraine and anxiety has been widely studied, given that migraineurs are more prone to suffer from anxiety [44]. On the other hand, we did not find any association between previous vascular disorders and fluctuations of the tinnitus on one side and THI score on the other. In our opinion, in subjects with a stable audiometric threshold, fluctuation may be related with daily anxiety level, although ambient noise and daily activities may play a role in it, since tinnitus is less perceived when paying an increased amount of attention to working activities.

Our work has several limitations. Firstly, since data have been collected from our records, we are unable to establish THI questionnaire subscales. Moreover, further studies with validated questionnaires are needed to correlate annoyance with the peculiar personality trait. Finally, our sample has some peculiarities, since in many cases they had other consultations before ours, and only “motivated” (with a high annoyance) subjects asked for evaluation in a specialized center. Finally, psychiatric comorbidity was only based on clinical history and no specific questionnaires were administered.

Nonetheless, our data are not inconsistent with the hypothesis that psychological disorders and a particular personality trait may be the main causal factors for tinnitus annoyance.

## Figures and Tables

**Table 1:** Values of the THI total score and grading in the subsamples of normoacousic subjects, with symmetric, asymmetric, and monolateral hearing loss. For the continuous variable (THI total score), an ANOVA for repeated measures was performed; for the other categorical variables (grading of THI score) a Χ^2^ test was performed.

	THI Total Score	Very Mild Tinnitus	Mild Tinnitis	Moderate	Severe	Catastrophic
**Normoacousic (n = 363)**	41.5 ± 23.5	62 (17.1%)	117 (32.2%)	74 (20.4%)	83 (22.8%)	27 (7.5%)
**Symmetric hearing loss (n = 468)**	43.8 ± 23.6	55 (11.7%)	168 (35.9%)	119 (25.5%)	84 (18%)	42 (8.9%)
**Asymmetric hearing loss (n = 185)**	41.9 ± 28.1	24 (13%)	64 (34.6%)	48 (25.9%)	36 (19.5%)	13 (7%)
**Monolateral hearing loss (n = 140)**	43.7 ± 24.7	20 (14.4%)	47 (33.7%)	35 (25.1%)	29 (20.7%)	9 (6.1%)
***p* value**	*p* = 0.52, F stat 0.74	*p* = 0.17 Χ^2^ = 5	*p* = 0.7 Χ^2^ = 1.2	*p* = 0.3 Χ^2^ = 3.5	*p* = 0.36 Χ^2^ = 3.1	*p* = 0.7 Χ^2^ = 1.4

**Table 2:** Linear regression model considering age, recognized causal factor, fluctuations of the tinnitus, migraine, clinical history of vascular disorders, and hearing loss as independent variables, and THI total score as a dependent variable.

Term	Coefficient	95% CI		t Stat	*p*
**Age**	0.1491	0.0052	0.2929	2.03	0.0423
**Recognized causal factor**	−1.203	−4.447	2.040	−0.73	0.4666
**Fluctuations**	2.725	−0.896	6.346	1.48	0.1400
**Psychiatric comorbidities**	14.35	9.83	18.86	6.24	<0.0001
**Migraine**	5.462	0.605	10.319	2.21	0.0276
**Vascular disorders**	0.636	−2.130	3.402	0.45	0.6518
**Hearing loss**	0.09396	3.36235	3.55026	0.05	0.9574

**Table 3:** Grading of the annoyance of tinnitus in the subgroup of subjects with and without previous psychiatric diagnoses (chi-square statistic is 47.6, *p*-value is <0.00001). Between parentheses is the percentage of the total number of subjects in each subsample.

	Psychiatric (n = 218)	Non Psychiatric (n = 938)
**Very Mild (0–18)**	8 (3.2%)	48 (5.1%)
**Mild (18–36)**	40 (18.4%)	370 (39.4%)
**Moderate (38–56)**	56 (25.8%)	216 (23.1%)
**Severe (58–76)**	65 (29.9%)	204 (21.7%)
**Catastrophic (78–100)**	49 (22.7%)	100 (10.7%)

## Data Availability

Data is available upon request.
